# Speciation of inorganic selenium and selenoamino acids by an HPLC-UV-HG-AFS system

**DOI:** 10.1155/S1463924601000207

**Published:** 2001

**Authors:** I. Ipolyi, W. Corns, P. Stockwell, P. Fodor

**Affiliations:** 1 Department of Applied Chemistry Szent Istvan University 29. Villanyi ut Budapest H- 1118 Hungary; 2 PS Analytical Ltd Arthur House, Unit 3 Crayfields Industrial Estate Main Road, St Pauls Cray, Orpington, Kent BR5 3HP UK

## Abstract

For the on-line speciation of selenocystine (SeCys), selenomethionine (SeMet), selenoethionine (SeEt), selenite (Se(IV)) and selenate (Se(VI)), a high-performance liquid chromatography-UV irradiation-hydride generation-atomic fluorescence spectro- metric method is described. Separation was carried out on a conventional reversed-phase C18 column modified with didodecyl- dimethylammonium bromide with gradient elution applying two concentrations of ammonium acetate as the mobile phase. UV irradiation and hydride generation parameters were optimized. The obtained detection limits for SeCys, SeMet, SeEt, Se(IV) and Se(VI) were 0.31, 0.43, 0.7, 0.44 and 0.32 ng ml^−^1, respectively, using a 100-wl loop. The method was tested with spiked mineral water and two volunteers' urine samples.


Journal of Automated Methods & Management in Chemistry, Vol. 23, No. 6 (November–December 2001) pp. 167–172

### Speciation of inorganic selenium and selenoamino acids by an HPLC-UV-HG-AFS system

**I. Ipolyi[1,2], W. Corns[3], P. Stockwell[3] and P. Fodor[1]**

[1] *Department of Applied Chemistry, Szent Istvan University, 29. Villanyi ut, H-1118, Budapest, Hungary;* [2] *e-mail: iipolyi@omega.kee.hu;* [3] *PS Analytical Ltd, Arthur House, Unit 3, Crayfields Industrial Estate, Main Road, St Pauls Cray, Orpington, Kent BR5 3HP, UK*

*For the on-line speciation of selenocystine (SeCys), selenomethionine (SeMet), selenoethionine (SeEt), selenite (Se(IV)) and selenate (Se(VI)), a high-performance liquid chromatography-UV irradiation-hydride generation-atomic fluorescence spectrometric method is described. Separation was carried out on a conventional reversed-phase C18 column modified with didodecyldimethylammonium bromide with gradient elution applying two concentrations of ammonium acetate as the mobile phase. UV irradiation and hydride generation parameters were optimized. The obtained detection limits for SeCys, SeMet, SeEt, Se(IV) and Se(VI) were 0.31, 0.43, 0.7, 0.44 and 0.32 ng ml$^{-1}$, respectively, using a 100-μl loop. The method was tested with spiked mineral water and two volunteers' urine samples.*

### Introduction

Selenium is both an essential and toxic element and it has the narrowest tolerance window of any element [1]. It has been the most controversial element of the 20th century. In the first half of the century, it was treated as highly toxic and carcinogenic, but from the 1960s it was essential and anti-carcinogenic.

Selenium species are widely distributed in the environment and in living organisms. The inorganic species, selenite and selenate, are mainly present in waters and soils. The volatile organoselenium compounds, such as dimethyl selenide and dimethyl diselenide, are the result of biomethylation of inorganic selenium by microorganisms. More complex forms, such as the selenoamino acids selenocystine and selenomethionine, are found in various biological tissues as a result of metabolic pathways by which selenium is incorporated into proteins [2–4]. Most importantly, it occurs in humans in the enzyme gluthatione peroxidase, which protects cells against oxidative damage. Since oxidative damage is known to launch on the formation of various types of cancer [5], selenium has been attracting a great deal of attention. Several studies have indicated that it is of fundamental importance in which form selenium is introduced to the body because the inorganic forms are excreted directly, while the organoselenium compounds are retained by the body. Since most EU countries have selenium dietary levels well below WHO guidelines, supplements are being produced [6]. Several selenium food supplements are found on the market, but with controversial organic and inorganic selenium content. Therefore, there is an increasing need for selenium speciation methods that not only the well-supplied research laboratories can afford.

Analytical systems developed for selenium speciation employ a powerful high-performance liquid chromatography (HPLC) coupled to a specific atomic detector with a high efficiency sample introduction system.

Fast and efficient separation of the inorganic from the organic selenium species can be solved microcolumns [7]. Different selenoamino acids and the inorganic forms (selenite and selenate) can be separated on cation-exchange, strong anion-exchange and reversed-phase columns [8–13]. The separation of selenoamino acids, the inorganic forms and two other species, selenourea and trimethylselenonium, can be performed on a reversed-phase C-18 column modified with didodecylammonium bromide (DDAB) [14]. The chiral separation of selenoamino acid enantiomers has been achieved by crown ether and teichoplanin-based chiral stationary phases [15, 16].

The most favoured detector to date for selenium speciation is inductively coupled plasma mass spectrometer (ICP-MS) coupled directly or with a high-efficiency nebulization to HPLC. Although ICP-MS offers very low detection limits in general, the elemental sensitivity of Se is distributed over six natural isotopes and the most abundant ones suffer from strong spectral interferences. Another approach employs an intermediate step, microwave digestion (MW) or UV irradiation, together with a redox reagent, followed by hydride generation (HG) an element specific detection, atomic absorption or atomic fluorescence [17–21]. The intermediate step ensures the destruction of the organic part of the organoselenium molecules and reduction of all the species to selenite before HG.

In this paper, a relatively inexpensive but robust procedure is proposed for the speciation of SeCys, SeMet, SeEt, Se(IV) and Se(VI). An ion-pair chromatographic method is followed by on-line heating and UV irradiation for the breakdown and reduction of the species to Se(IV) with the mixture of HCl + KBr, then the generated hydrides are determined by atomic fluorescence spectrometry. The procedure has been used for the analysis of water and urine samples.

### Experimental

### *Reagents*

All reagents were of analytical reagent grade. All aqueous solutions were prepared using deionized water (Elga Ltd, High Wycombe, UK). Seleno-DL-methionine,

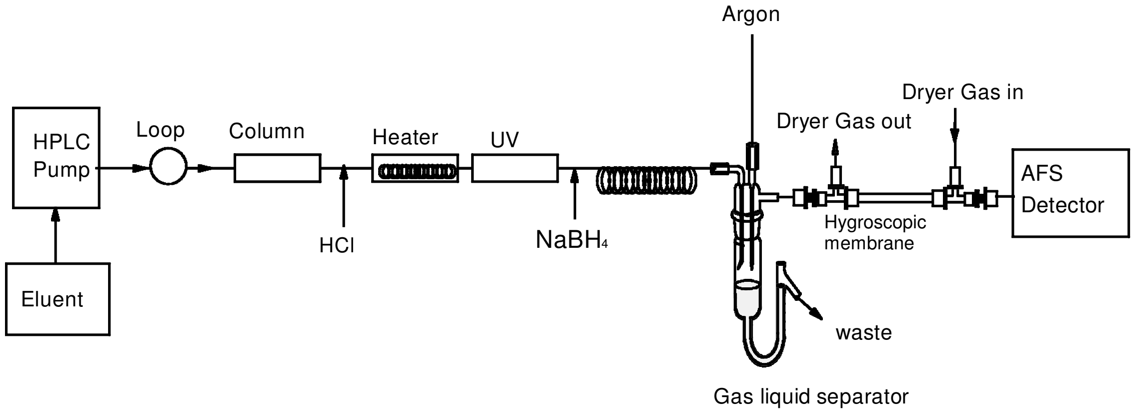

*Figure 1. Schematic of the developed system.*

seleno-DL-ethionine and seleno-DL-cystine were purchased from Sigma Chemical Co. (St Louis, MO, USA); $Na_2SeO_4$ was from Sigma (Gillingham, UK). Se(IV) 1000 mg $l^{-1}$ stock solution was obtained from Merck (Darmstadt, Germany). Sodium borohydride, sodium hydroxide, hydrobromic acid, potassium bromide and ammonium acetate were purchased from Aldrich; hydrochloric acid from Analar; methanol from Merck; and the DDAB ion-pairing agent from Sigma.

*Instrumentation*

A Merck 7100 four-channel HPLC pump and a six-port injection valve (Rheodyne, CA, USA) with a 100 μl loop were connected to a DDAB modified RP-18-bonded silica stationary phase (Merck, Darmstadt, Germany). A manually controlled electrical switch-valve was used to perform the gradient elution program. For detection, a Millennium Excalibur AFS detector (PS Analytical, Orpington, UK) equipped with a Se-boosted discharge hollow cathode lamp (Photron, Victoria, Australia) was used. The Millennium Excalibur System was supplied with two peristaltic pumps and a gas–liquid separator (GLS) required for hydride generation. Argon was the carrier gas in the GLS. It was dried with a hygroscopic membrane drying tube (Perma Pure Products, Farmingdale, NJ, USA) and air was used as the drying gas at a flow rate of 2.5 l $min^{-1}$. The on-line prereduction step was performed with a heating jacket and a low-pressure mercury lamp (TNN 15/32, 15 W; Heraeus, Germany). Signal output was recorded with either a potentiometer chart recorder or Borwin chromatographic software. The system is shown in figure 1.

*Procedure*

A total of 100 μl sample solution was injected on the HPLC column and selenium species were separated using a gradient elution program. Acid solution was added after separation. Then the species were subjected to prereduction by the heating jacket adjusted to 200°C followed by the UV-cracking system, in all together 12 m of 0.8 mm i.d. PTFE tubing. Then the mixture was cooled in an ice-bath, in a 1 m, 0.8 mm i.d. PTFE tubing to minimize water vapour transfer from the GLS to the detector. Hydride generation was carried out by adding the borohydride solution. The selenium hydrides then passed to the GLS where the argon flow entered to transfer them to the Ar-$H_2$ cold flame of the detector through the hygroscopic membrane drying tube. A BDHCL was used as the excitation source. All data were calculated by considering both peak heights and peak areas. Working parameters are shown in table 1.

### Results and discussion

*Chromatographic separation*

The separation of the five species was carried out on a DDAB-modified C18-bonded silica column. The column was modified by passing $10^{-2}$ mol $l^{-1}$ DDAB in a water–methanol (1:1) solution through the column at a flow rate of 1 ml $min^{-1}$ for 2 h.

For the development of the gradient elution program, the elution program applied to the separation of the selenoamino acids and Se(IV) [22] was used as a starting point. It used 0.01 mol $l^{-1}$ ammonium acetate with 0.5% (v/v) MeOH and $10^{-5}$ mol $l^{-1}$ DDAB content.

To obtain baseline separation of SeCys and SeMet, 0.008 mol $l^{-1}$ ammonium acetate at a flow rate of 0.8 ml $min^{-1}$ proved the best. At either higher concentrations or higher flow rates, the separation was not complete, while at smaller concentrations and smaller flow rates considerable peak broadening was observed. For the fast elution of Se(VI), more concentrated ammonium acetate was required. A total of 0.2 mol $l^{-1}$ ammonium acetate conducted at 1.5 ml $min^{-1}$ ensured a 15-min complete runtime. Se(IV) can be eluted with 0.008 mol $l^{-1}$ mobile phase, but with a serious tailing. Therefore, changing from mobile phase 1 to mobile phase 2 and from flow rate 0.8 ml $min^{-1}$ to 1.5 ml $min^{-1}$ at 7.2 min, the separation of the five species can be achieved in 15 min.

*Preredution conditions*

The optimization of prereduction conditions was started with the parameters proposed by Vilano and Rubio [21] for the Hereaus TNN 15/32 low-pressure Hg vapour lamp with 50% (v/v) HCl and 120 s irradiation time. No signal was obtained for Se(VI) under the circum-

*Table 1. Optimal working parameters of the system.*

| | |
|---|---|
| HPLC parameters | |
| Column | LiChroCART® 124-4, 5 μm LiChrospher® 100 RP-18 |
| Mobile phase 1 | 0.08 mol $l^{-1}$ ammonium acetate, pH = 4, 0.5% (v/v) MeOH $10^{-5}$ mol $l^{-1}$ DDAB |
| Mobile phase 2 | 0.2 mol $l^{-1}$ ammonium acetate, pH = 7, 0.5 (v/v)% MeOH $10^{-5}$ mol $l^{-1}$ DDAB |
| Injected volume | 100 μl |
| Gradient elution | 0–7.2 min: 0.08 mol $l^{-1}$ mobile phase 1; 0.8 ml $min^{-1}$<br>7.2–15 min: 0.2 mol $l^{-1}$ mobile phase 2; 1.5 ml $min^{-1}$ |
| Prereduction | |
| Reducing agent | 50% (v/v) HCl—5% (m/v) KBr; 3 ml $min^{-1}$ |
| Heating | 200°C |
| UV source | low pressure mercury lamp, 15 W |
| HG parameters | |
| Acid solution | 50% (v/v) HCl—5% (m/v) KBr; 3 ml $min^{-1}$ |
| Reducing agent | 1% (m/v) $NaBH_4$ in 1% (m/v) NaOH: 3 ml $min^{-1}$ |
| Ar flow rate | 300 ml $min^{-1}$ |
| AFS parameters | |
| Primary current | 25 mA |
| Boost current | 25 mA |

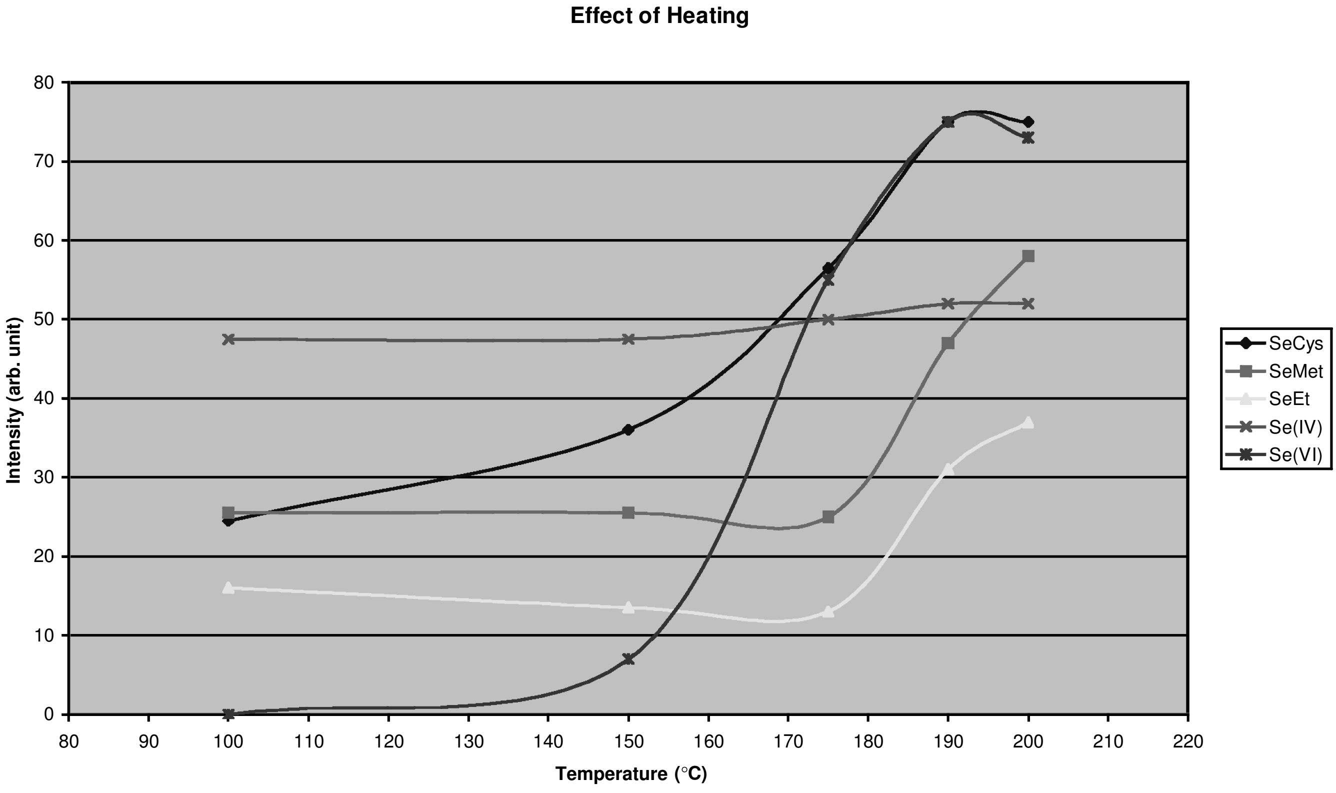

*Figure 2. Effect of changing the temperature of the heating jacket on the signal-to-background ratio of the five investigated species.*

stances provided by our laboratory. $H_2SO_4$ and HBr were also tried, but without success. Then the heating jacket was included in the system before the UV cracker. The heating temperature was optimized in the 80–220°C range and the signal-to-background ratio (SBR) proved to be the highest at 200°C (figure 2). SBR decreases both below and above this temperature. Below 200°C, the net signal decreases to a higher extent than the baseline noise, while above this temperature, the baseline noise increases enormously. The actual temperature in the PTFE tubing could not be checked, but bubble formation that may disturb the analysis was avoided.

The efficiency of the system was checked with heating, but switching off the UV cracker. This combination caused the decrease of the selenoamino acid signals to 10–15% and the signal of Se(VI) to 90%. This observation suggests that the major role of UV irradiation in the system was the breakdown of organoselenium compound to inorganic Se, while the heated reducing mixture of

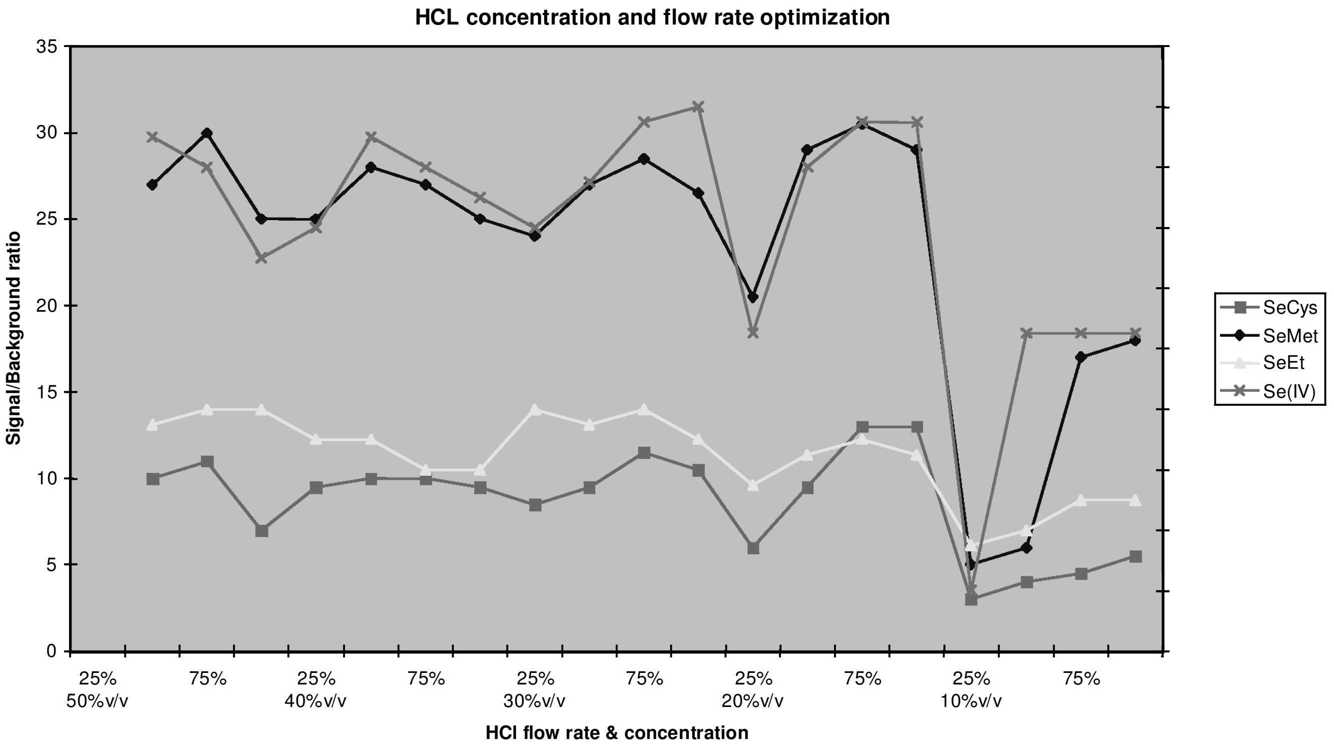

*Figure 3. Effect of changing the concentration and flow rate of hydrochloric acid on the signal-to-background ratio of selenocystine, selenomethionine, selenoethionine and Se(IV).*

HCl/KBr carried out the transformation of Se(VI) to Se(IV).

### *Derivatization optimization*

The hydride generation step was optimized by changing the concentration and flow rate of HCl and $NaBH_4$, one parameter at a time.

The range of flow rate optimization for both reagents was 1–5 ml $min^{-1}$. The concentration of HCl was varied between 10 and 50% (v/v). The borohydride concentration was optimized between the value recommended by the manufacturer for total Se measurement with the AFS detector and the optimum value gained for the direct hydride generation of selenoamino acids and Se(IV) [22], 0.7% (m/v) $NaBH_4$ in 0.1 mol $l^{-1}$ NaOH to 4% (m/v) $NaBH_4$ in 0.5 mol $l^{-1}$ NaOH.

Optimization was carried out for the species that form hydride directly (SeCys, SeMet, SeEt, Se(IV)) without heating and UV irradiation, and then for the proposed five species including Se(VI) with heating and UV irradiation. In case of direct hydride generation, the highest SBR was obtained for 20% (v/v) HCl conducted at 3 ml $min^{-1}$ with 1% (m/v) $NaBH_4$ in 0.5% (m/v) NaOH. Figure 3 shows that the effect of flow rate change is more pronounced at this concentration range than the effect of varying the concentration. Including the pre-reduction step in the system, the hydride generation parameters had to be re-optimized. This set-up, or rather the SBR of Se(VI), requires 50% (v/v) HCl and the presence of KBr. The re-optimization was carried out with a 20–50% (v/v) HCl and 2.5–10% (m/v) KBr mixture, where the 50% (v/v) HCl–5% (m/v) KBr mixture proved the most suitable. The borohydride concentration was maintained because if it was increased, after a temporary increase of SBR for all species, the flame background increased, therefore SBR decreased and, after a few runs, moisture plugged the way of hydrides to the detector.

### *Quality parameters of the method*

The linear range was established primarily by peak height measurements. The validity of peak height measurements was checked at different concentrations both in the standard solution and in samples with the standard addition by peak area determinations. All the studied Se species showed a three orders of magnitude linear range from the detection limit.

Precision was calculated as the RSD of nine peak height measurements for all the five species at 10 μg $l^{-1}$.

Detection limits were calculated at a concentration of 1 μg $l^{-1}$ for all the five species from nine replicates, as 3 × SD ($\sigma_n - 1$). The DL obtained for SeCys, SeMet, SeEt, Se(IV) and Se(VI) were 0.31, 0.43, 0.7, 0.44 and 0.32 μg $l^{-1}$, respectively.

The comparability of the obtained analytical parameters with results gained by other selenium speciation systems is shown in table 2. It clearly shows that the newly developed HPLC-UV-HG-AFS system gives better detection limits than the presented systems; and that the detection limit of Se(VI) was dramatically improved compared with the other approaches using hydride generation.

*Table 2. Analytical parameters of the developed system in comparison with the analytical parameters of other selenium speciation systems.*

| | Limit of detection ($\mu g l^{-1}$) | | | | |
|---|---|---|---|---|---|
| Se species | HPLC-HG-AFS[1] | HPLC-HG-AFS[2] | HPLC-Heating-UV-AFS[2] | LC-UV-HG-AFS[3] | HLPC-MW-HG-ICP-MS[4] |
| SeCys | 18 (2.8%)* | 3.02 (4.1%) | 0.31 (2.2%) | 0.9 (1.1%) | 0.7 (2%) |
| SeMet | 70 (3.1%) | 0.75 (2.2%) | 0.43 (3.4%) | 5.9 (4.9%) | 1.2 (2%) |
| SeEt | 96 (3.7%) | 1.03 (1.5%) | 0.70 (9.7%) | — | 4.4 (2%) |
| Se(IV) | 160 (2.9%) | 0.36 (3.1%) | 0.44 (4.4%) | 0.6 (3.3%) | 2.0 (2%) |
| Se(VI) | — | — | 0.32 (2.5%) | 14.5 (3.6%) | 1.7 (2%) |

* RSD (%) are in parentheses.

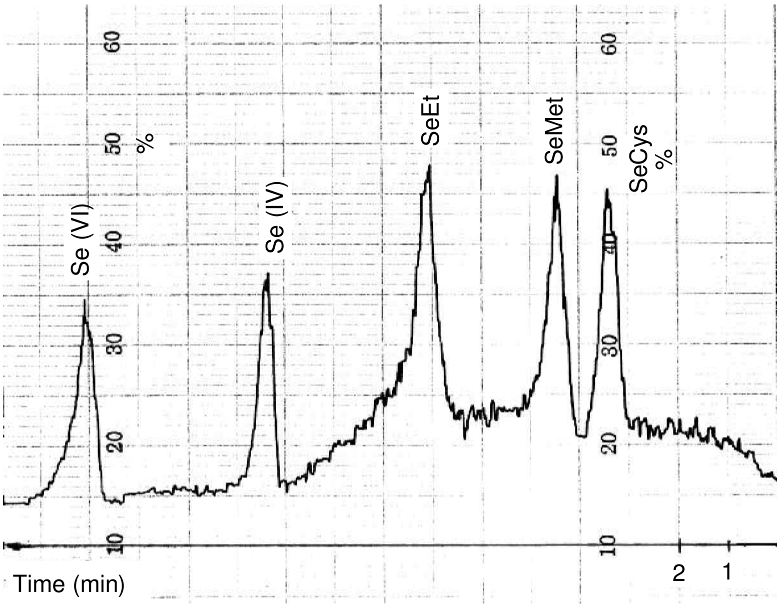

*Figure 4. Chromatogram of the spiked mineral water with 10 μg $l^{-1}$ addition for all the five species.*

*Analysis of samples*

The developed method was used to analyse a bottled mineral water and two volunteers' urine samples.

The analysis of mineral water revealed the absence of selenium species. Therefore, a two-step standard addition was carried out to check the recovery of the method and the possible matrix interferences. For both 5 and 10 $\mu g l^{-1}$ addition of all species, recoveries were between 96 and 104%. The matrix did not modify the retention time of the species, which means that the chromatogram of the 10 $\mu g l^{-1}$ standard addition of the mineral water sample corresponded to the chromatogram of a standard solution containing 10 $\mu g l^{-1}$ of all the five species (figure 4).

The volunteers' urine samples was taken 12 h after the consumption of a reasonable amount of Brazil nuts, which are known to have a high Se content.

In the analysis, three peaks were detected, SeCys, SeMet and a third peak that could not be identified. It is supposed that the unidentified peak, which is the largest of all, is trimethylselenonium, since this form accounts for a high per cent of Se in urine especially after a large nutritional intake [23]. A representative chromatogram of a 1:5 dilution of one of the urine samples obtained by the method proposed here is shown in figure 5. The identities of the SeCys and SeMet were controlled by standard addition. The spiked concentration for both SeCys and SeMet was about the same originally contained by the sample. A good recovery of the spiked species was observed: $94.9 \pm 5.0\%$ for SeCys, $96.6 \pm 5.8\%$ for SeMet, $97.2 \pm 4.6\%$ for Se(IV), $103.6 \pm 3.4\%$ for Se(VI) and $98 \pm 1.2\%$ for total selenium content.

### Conclusion

The main advantage of the method presented here for selenium speciation is that it requires a powerful, but relatively inexpensive, instrument combination compared with other element selective atomic detectors. At the same time, the analytical parameters obtained with the system render the analysis of real samples possible. In a forthcoming work, other chromatographic methods will be applied in the system for the validation of chromatography presented here.

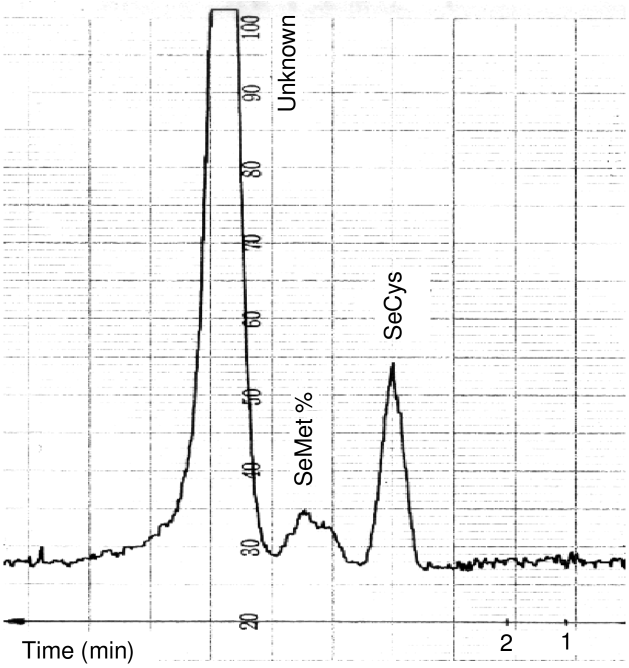

*Figure 5. Representative chromatogram of the 1:5 dilution of a urine sample with identified SeCys and SeMet peaks.*

### Acknowledgement

The financial support of OTKA under Grant Nos FO 32296 and TO 29040 is greatly acknowledged. The authors are very grateful to Professor Stockwell for the opportunity to carry out the main part of the work in the laboratory of PS Analytical.

### References

1. SEAS Project.
2. Jiang, S., Robberecht, H., Adams, F. and Vanden Berghe, D., *Toxicological Environmental Chemistry*, **6**, 1983, 191.
3. Bonelli, L., Conio, M. and Massa, P., *Cancer Prevention and Control*, **100**, 1998, A351.
4. Rayman, M. P., *British Medical Journal*, **314**, 1997, 387.
5. Combs, G. F. and Clark, L., *Selenium and Cancer Prevention: Antioxidant Nutrients and Disease Prevention* (Boca Raton: CRC Press, 1997).
6. Ebdon, L., *Speciation*, **2**, 2000, 21.
7. Fitzpatrick, S. A., Ebdon, L. and Foulkes, M., 4th Euroconference on Environmental Analytical Chemistry, *Proceedings*, **128**, 2000.
8. Lei, T. and Marshall, W. D., *Applied Organometallic Chemistry*, **9**, 1995, 149.
9. Thomas, C., Jakubowski, N., Stuewer, D., Klockow, D. and Emons, H., *Journal of Analytical Atomic Spectrometry*, **13**, 1998, 1221.
10. Goessler, W., Kuehnelt, D., Schlagenhaufen, C., Kalcher, K., Abegaz, M. and Irgolic, K. J., *Journal of Chromatography A*, **789**, 1998, 233.
11. Alsing Pedersen, G. and Larsen, E. H., *Fresenius Journal of Analytical Chemistry*, **358**, 1997, 591.
12. Uden, P. C., Bird, S. M., Kotrebai, M., Nolibos, P., Tyson, J. F., Block, E. and Denoyer, E., *Fresenius Journal of Analytical Chemistry*, **362**, 1998, 447.
13. Casiot, C., Szpunar, J., Lobinski, R. and Potin-Gautier, M., *Journal of Analytical Atomic Spectrometry*, **14**, 1999, 645.
14. González LaFuente, J. M., Dlaska, M., Fernández Sánchez, M. L. and Sanz-Medel, A., *Journal of Analytical Atomic Spectrometry*, **13**, 1998, 423.
15. Ponce de Leon, C. A., Sutton, K. L., Caruso, J. A. and Uden, P. G., *Journal of Analytical Atomic Spectrometry*, **15**, 2000, 1103.
16. Pérez Méndez, S., Blanco González, E. and Sanz-Medel, A., *Journal of Analytical Atomic Spectrometry*, **15**, 2000, 1109.
17. Gómez-Aria, J. L., Sánchez-Rodas, D., Morales, E., Hergott, O. and Marr, I. L., *Applied Organometallic Chemistry*, **13**, 1999, 783.
18. Pitts, L., Fisher, A., Worsfold, P. and Hill, S. J., *Journal of Analytical Atomic Spectrometry*, 1995, 519.
19. Rubio, R., Padró, A. and Rauret, G., *Analytica Chimica Acta*, **353**, 1997, 91.
20. Vilanó, M., Padró, A., Rubio, R. and Rauret, G., *Journal of Chromatography A*, **819**, 1998, 211.
21. Vilanó, M. and Rubio, R., *Journal of Analytical Atomic Spectrometry*, **15**, 2000, 177.
22. Ipolyi, I., Stefánka, Zs. and Fodor, P., *Analytica Chimica Acta*, 2001 (in press).
23. Lobinski, R., Edmonds, J. S., Suzuki, K. T. and Uden, P. C., *Pure Applied Chemistry*, **72**, 2000, 447.

